# Increase of 5-HT levels is induced both in mouse brain and HEK-293 cells following their exposure to a non-viral tryptophan hydroxylase construct

**DOI:** 10.1038/s41398-021-01634-x

**Published:** 2021-10-08

**Authors:** Emiliano Tesoro-Cruz, Leticia Manuel-Apolinar, Norma Oviedo, Sandra Orozco-Suárez, Minerva Crespo Ramírez, Vilma Carolina Bekker-Méndez, M. Magdalena Aguirre-García, Sandra Angélica Rojas-Osornio, Vladimir Paredes-Cervantes, Miguel Pérez de la Mora

**Affiliations:** 1 Unidad de Investigación Biomédica en Inmunología e Infectología, Hospital de Infectología, Centro Médico Nacional “La Raza”, IMSS, Ciudad de México, México; 2 Unidad de Investigación Médica en Enfermedades Endócrinas, UMAE, Hospital de Especialidades, Centro Médico Nacional “Siglo XXI”, IMSS, Ciudad de México, México; 3 Unidad de Investigación Médica en Enfermedades Neurólogicas, UMAE, Hospital de Especialidades, Centro Médico Nacional “Siglo XXI”, IMSS, Ciudad de México, México; 4 División de Neurociencias, Instituto de Fisiología Celular, Universidad Nacional Autónoma de México, Ciudad de México, México; 5 Unidad de Investigación UNAM-INC, División de Investigación, Facultad de Medicina, UNAM, Instituto Nacional de Cardiología Ignacio Chávez., Ciudad de México, México; 6 Sección de Estudios de Posgrado e Investigación de la Escuela Superior de Medicina del Instituto Politécnico Nacional, Ciudad de México, México

**Keywords:** Depression, Molecular neuroscience

## Abstract

Tryptophan hydroxylase type 2 (Tph2) is the rate-limiting enzyme for serotonin (5-HT) biosynthesis in the brain. Dysfunctional Tph2 alters 5-HT biosynthesis, leading to a deficiency of 5-HT, which could have repercussions on human behavior. In the last decade, several studies have associated polymorphisms of the *TPH2* gene with suicidal behavior. Additionally, a 5-HT deficiency has been implicated in various psychiatric pathologies, including alcoholism, impulsive behavior, anxiety, and depression. Therefore, the *TPH2* gene could be an ideal target for analyzing the effects of a 5-HT deficiency on brain function. The aim of this study was to use the construct pIRES-hrGFP-1a-Tph2-FLAG to treat CD1-male mice and to transfect HEK-293-cells and then to evaluate whether this treatment increases 5-HT production. 5-HT levels were enhanced 48 h post-transfection, in HEK-293 cells. Three days after the ocular administration of pIRES-hrGFP-1a-Tph2-FLAG to mice, putative 5-HT production was significantly higher than in the control in both hypothalamus and amygdala, but not in the brainstem. Further research will be needed on the possible application of this treatment for psychiatric diseases involving a Tph2 dysfunction or serotonin deficiency.

## Introduction

Stress and affective disorders, including anxiety and depression, can have severe consequences. Depression affects 350 million people around the world and is the leading cause of disability in terms of total lost working years [1]. By 2030, depression is estimated to become the second cause of morbidity in middle-income countries and the third one in low-income ones [1, 2].

Depression is a mental illness manifested as a mood disorder, which is characterized by a decreased level of energy and typically involves low self-esteem and a loss of interest or pleasure (anhedonia) in activities otherwise considered entertaining. It is suggested that a dysfunction of the serotonergic system is closely related to most mood disorders, including depression [2, 3]. Studies in rodents have emphasized the importance of this neuron system in stress [3] and in the pathophysiology of suicidal behavior [4, 5]. Indeed, neuromodulators have been in the last years the main targets in biological psychiatry for the treatment of such disorders [6].

In the two-step process of serotonin (5-HT) biosynthesis, the first and rate-limiting step is the conversion of the amino acid l-tryptophan into 5-hydroxytryptophan (5-HTP), a reaction catalyzed by the activity of the enzyme tryptophan hydroxylase (TPH) [7–9]. Two TPH genes have been described, *TPH1* and *TPH2*. The former is primarily located in a variety of non-neuronal cells, such as the enterochromaffin cells of the gut and the pineal gland [10–12], while the latter is expressed in the intestinal myenteric plexus and within the serotonergic neurons in the raphe nuclei [13].

Since the *TPH2* gene encodes the enzyme for the rate-limiting step of 5-HT biosynthesis in the brain, is an ideal target for experiments aimed at exploring the effect of a 5-HT deficiency on brain homeostasis [13]. This deficiency is reportedly related to alterations in the TPH2 protein or *TPH2* gene. For instance, a close connection between diminished TPH2 activity and depression [14, 15] has been detected based on single nucleotide polymorphisms (SNPs). In addition, different *TPH2* polymorphisms have been linked to suicidal behavior and depressive disorders [16–20], and haplotype analysis of the *TPH2* gene has revealed evidence of variants associated with depression [21], suicide [12, 20], and bipolar affective disorder [22].

Gene therapy can be provided to correct a deficiency resulting from altered or absent genes. It is carried out by the transfer of genes to modulate cell function and modify protein expression. However, there are certain risks involved in this therapy, including those implicit in long-term gene expression generated by viral vectors. Moreover, the duration of expression must be appropriate to treat the intended disease. Consequently, transient expression with a non-viral vector seems a promising approach to treat brain diseases caused by serotonin deficiency.

The greatest challenge in gene therapy is to find efficient vectors for transferring genes to internal structures of the brain. The eye is a possible target for gene therapy in brain diseases due to its accessibility to the brain and its privileged immune characteristics that preclude inflammatory and immune reactions against non-viral vectors [23, 24]. Recently, our group has demonstrated that a novel non-viral Tph2 construct (pIRES-hrGFP-1a-Tph2-FLAG) administrated by the ocular via is capable of reaching the brain. Additionally, it was also showed that such a plasmid was transcribed and translated both in vitro and in vivo [25]. The aim of the present study was to examine whether the construct pIRES-hrGFP-1a-Tph2-FLAG could be expressed and increases 5-HT production both in HEK-293 cells and in mice after its ocular administration.

## Methods

### pIRES-hrGFP-1a-Tph2-FLAG and plasmid purification

The preparation of the novel non-viral Tph2 construct (pIRES-hrGFP-1a-Tph2-FLAG) was accomplished using the plasmid pIRES-hrGFP-1a (pIRES, 240031, Agilent Technologies, La Jolla, CA, USA), in which the murine *Tph2* gene is fused into the FLAG sequence (DYKDDDDK) frame, as previously reported [25]. Plasmids were grown in *Escherichia coli* DH5α, which was used as the recipient of plasmids for amplification. Plasmid purification was performed using a commercial kit (Plasmid Plus Maxi Kit, 12965, QIAGEN, Maryland, USA), as specified in the manufacturer’s instructions.

### Cultures of the kidney cell line (HEK-293)

The human embryonic kidney cell line (HEK-293) from ATCC (HEK-293, CRL-1573) is easy to grow and transfect, and thus, it commonly serves as a model for examining gene expression. In the current study, these cells were used for transfection followed by an analysis of the in vitro putative 5-HT production. HEK-293 cells show an expression pattern typically similar to that observed in neural cells [26], showing the presence of neurofilament proteins, 5-HT transporter (SERT), 5-HT (6/7) receptors, and Tph2 [26, 27].

HEK-293 cells were cultured in Dulbecco’s modified Eagle medium (DMEM) supplemented with 10% fetal bovine serum (FBS) and incubated at 37 °C under 90% relative humidity and 5% CO_2_ atmosphere. HEK-293 (1.35 × 10^6^) cells were seeded in 10 cm plates. 24 h later the cultures having a cellular confluence of 70–80% were transfected. Transfection was performed with 20 μL of TurboFect Transfection Reagent (TurboFect™ Transfection Reagent, R0532, Thermo Fisher Scientific, MA, USA) and 10 μg of the DNA plasmid per plate, which was previously incubated for 15 min at room temperature. Subsequently, the DNA–TurboFect complex was slowly added to the HEK-296 cells followed by incubation for 48 h. Cells were harvested and frozen keeping them at −20 °C until their further use in enzyme-linked immunosorbent assays (ELISA). 5-HT quantitation was carried out in pIRES-hrGFP-1a-Tph2-FLAG transfected cells and as well as in cells from three different negative controls: non-transfected cells, cells transfected with the vector only (pIRES-hrGFP-1a) or transfected with the vector plus Tph2 (pIRES-hrGFP-1a-Tph2).

### RNA folding analysis

In order to understand differences between TpH2 and Tph2-FLAG from the point of view of protein RNA folding, we performed RNA folding analysis using a server from the Theoretical Chemistry Institute, University of Vienna (http://rna.tbi.univie.ac.at/cgi-bin/RNAWebSuite/RNAfold.cgi). The cloned sequences from the murine Tph2 ORF and the Tph2-FLAG ORF were analyzed for the minimum free energy (MFE). The partition function calculation was obtained from the thermodynamic ensemble prediction. Thermodynamic parameters were registered and the MFE secondary RNA structure plot was selected with aid of the nucleotide sequence and base-pair probabilities.

### Animals

Healthy adult male CD1 mice, 10–12 weeks old (*n* = 24) weighing 35–40 g, were obtained from the local colony of the Instituto de Fisiología Celular, Universidad Nacional Autónoma de México. They were kept under controlled conditions housed in filter-top cages on a 12 h light/dark cycle (lights on at 7:00 h, at a temperature of 20 ± 1 °C, and with food and water ad libitum). The investigator was blinded to the mice groups allocation and when assessing the outcome during all experiments. Animals were treated in strict accordance with the Mexican Official Norm (the Guide for the Care and Use of Laboratory Animals, NOM-062-ZOO-1999). The protocol was approved by the Ethics in Animal Experiments Committee at the Instituto de Fisiología Celular, UNAM (number 2017- MPM123-17).

Mice were randomly divided into two groups, each given a different treatment (*n* = 12 each): 25 μL of PBS (control), and 25 μg of pIRES-hrGFP-1a-Tph2-FLAG diluted in 25 μL of PBS (experimental group). The way in which mice were assigned to the groups was by performing a simple random selection, where each mouse was taken and introduced into a cage (*n* = 6), in which all the elements that are part of a universe had the same probability of being selected.

PBS and the plasmid were administered to mice by placing, with the aid of a micropipette, 25 μL drops in both eyes. On day 0, nine mice from the control group were sacrificed to perform ELISA and three for immunohistochemistry assays. Three days after treatment, mice from the plasmid-treated group were sacrificed and distributed in the same way as above.

### 5-HT biosynthesis in vitro and in vivo

To evaluate the putative 5-HT biosynthesis in vitro, HEK-293 cells were cultured under the conditions previously described. Cells were incubated for 2 h in ice and centrifuged for 20 min at 10,000 rpm in a refrigerated microcentrifuge. Pellets were resuspended and lysed in 200 μL 1× RIPA Buffer (pH 7.5): 20 mM Tris–HCl, 150 mM NaCl, 1 mM Na_2_EDTA, 1 mM EGTA, 1% NP-40, a protease inhibitor cocktail (Complete, EDTA-free, 11873 Roche, Mannheim, Germany) and 1 mM PMSF. Lysates were incubated for 1 h at room temperature and after centrifugation supernatants were recovered and frozen at −70 ºC until used for 5-HT quantification.

5-HT levels were also measured in the brain of mice ex vivo, performing all assays in triplicate. Separate samples of about 30 mg of tissue were taken from the hypothalamus, amygdala, and brainstem after their dissection. Tissue samples were rinsed in ice-cold PBS (pH 7.2), gently dried with PBS blotted filter paper to remove blood, and weighed. Brain tissue was homogenized in ice-cold PBS (1:10) with the aid of a Tissue Homogenizer (Potter–Elvehjem tissue grinders, Thomas Scientific, NJ, USA) and then sonicated at 4 °C (Fisherbrand™ Model 120 Sonic Dismembrator, Fisher Scientific, MA, USA) by using 2 cycles of 30 s each to break cell membranes. Homogenates were centrifugated for 15 min at 5000 rpm and the supernatants were removed and stored at −70 °C until needed.

### Immunohistochemical analysis

Three mice from each group were randomly selected for immunohistochemical studies. Brains were quickly removed and fixed in paraformaldehyde (4% in saline phosphate buffer) for 72 h; sucrose for 24 h, and cryoprotected with isopentane (Sigma-Aldrich, St. Louis, MO, USA), frozen on dry ice and stored at −80 °C for the immunochemistry techniques. Sagittal sections (12 μm thick) were obtained from the medium line using a cryostat (Leica GM, 1510S, Germany) and mounted onto poly-l-lysine-coated slides. The sections were washed in 0.12 M PBS (pH 7.2–7.6) and incubated for 30 min at room temperature in a blocking solution containing 1% normal horse serum (Normal Horse Serum Blocking Solution, S2000Vector Laboratories Inc., Burlingame, CA, USA) in 0.12 M PBS. Sections were incubated for 24 h at 4 °C with rabbit anti-FLAG (G0-2731, Sigma-Aldrich, MO, USA) and mouse anti-Tph2 (AMAB91108, Sigma-Aldrich, MO, USA) antibodies, both diluted in PBS (1:200). The sections were washed with 0.12 M PBS for 15 min and immediately incubated for 2 h at room temperature with a fluorochrome-conjugated secondary antibody diluted 1:200 in PBS:anti-mouse IgG Alexa 486 (A-21202, Invitrogen, Thermo Fisher Scientific) or anti-rabbi IgG Alexa 546 (A11035, Invitrogen, Thermo Fisher Scientific). Subsequently, nuclei were stained with Hoechst 33343 trihydrochloride (H3570, Invitrogen, Thermo Fisher Scientific) and washed with 0.12 M PBS for 10 min. Sections were gently dried and mounted with a coverslip having a drop of mounting medium (Vectashield Antifade Mounting Medium, H-1000, Vector Laboratories Inc., Burlingame, CA, USA).

### Confocal microscopy

Brain sections were observed using a Nikon Ti Eclipse inverted confocal microscope equipped with an A1 imaging system controlled by the proprietary software (NIS Elements v.4.50). Dyes were excited in a sequential mode with built-in laser lines. Fluorescence was read in the following ranges: 425–475 nm for Alexa 486, 400–460 nm for Hoechst 33343, and 570–620 nm for Alexa 546. Images were acquired at ×20 (dry, NA 0.8) or ×60 (oil immersion, NA 1.4) and evaluated with NIS Elements v.4.50.

### 5-HT measurement

5-HT levels were measured by ELISA (1 ng/mL sensitivity) in 50 µL supernatant samples from both HEK-293 cell lysates and tissue homogenates according to the supplier’s instructions (US Biological Life Sciences, Salem, MA, USA), with assays performed in triplicate. The serotonin concentration in tissue was expressed in μg/g.

### Statistical analyses

Data are presented as the mean of three assays ± SE. Statistical differences between two groups having independent means were calculated using the two-tailed Student’s *t*-test. Statistical differences between multiple groups were analyzed using one-way analysis of variance (ANOVA) followed when appropriate (*P* < 0.05) by the Tukey test as a post-hoc test. Statistical significance was set at *P* < 0.05. Statistical computations were performed using the Prisma 8.0 Graph Pad Software.

## Results

### 5-HT quantification in HEK-293 cells

In order to study the potentiality of the construct pIRES-hrGFP-1a-Tph2-FLAG to trigger the putative synthesis of an active Tph2 enzyme, capable to increase 5-HT production, the levels of this neurotransmitter were studied in vitro using HEK-293 cells.

**Fig. 1:**
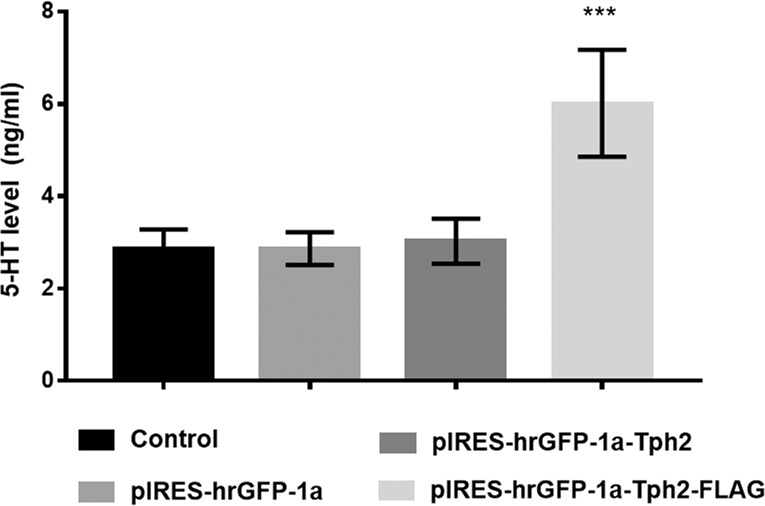
5-HT level in HEK-293 cells. Serotonin levels were measured in HEK-293 cells by ELISA at 48 h post-transfection. Evaluation was made in supernatants from lysates of both non-transfected (control) cells and from cells transfected with any one of the following three different treatments: pIRES (pIRES-hrGFP-1a), a commercial plasmid only having the vector; (pIRES-hrGFP-1a-Tph2), the murine Tph2 gene construct without FLAG and (pIRES-hrGFP-1a-Tph2-FLAG), the murine Tph2 gene construct with the FLAG tag. Cell lysates following transfection were centrifuged and their supernatants used to measure 5-HT. The statistical analysis shows a significantly greater 5-HT content in the lysates from cells treated with the construct pIRES-hrGFP-1a-Tph2-FLAG as compared to the lysates from the cells of all the other groups. Results are expressed as Means ± SEM, One-way ANOVA: F: 40.48, ***P < 0.001.

Global effects indicated that high 5-HT quantities were found (Fig. 1) in lysates of HEK-293 cells (One-way ANOVA: *F* = 40.48; *P* < 0.001). Post-hock analysis showed however that 5-HT levels were indeed higher in lysates from cells transfected with the pIRES-hrGFP-1a-Tph2-FLAG plasmid than those from nontransfected cells or transfected with either the commercial vector-only (pIRES-hrGFP-1a) or the construct with the murine *Tph2* gene added (pIRES-hrGFP-1a-Tph2) but without FLAG (*P* < 0.001; Tukey test).

### RNA folding analysis

**Fig. 2:**
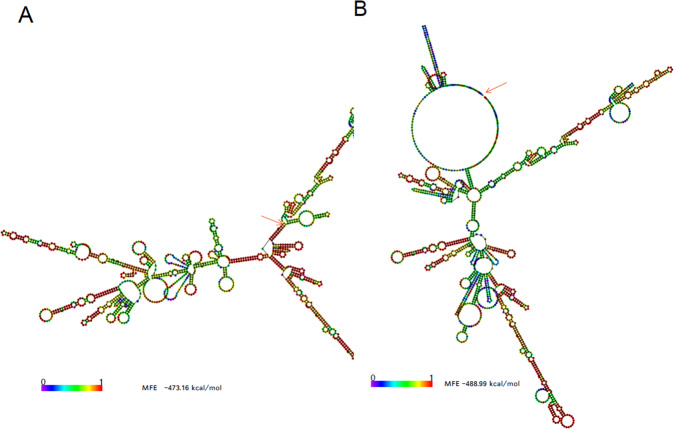
Tph2 RNA secundary structure. **A** Representation of the murine Tph2 mRNA folding showing its wild type 3'end. A red arrow points out to the start codon in the 5' end. The start codon is structured in a stem that could prevent its proper translation into protein. **B** Tph2-FLAG mRNA secondary structure shows the modified 3' end by the fusion of two FLAG sequences, which breaks the stem structure that contained the 5' AUG codon. A red arrow indicates the 5' end of the molecule that allows the start codon to be accessible to the ribosomes. Color gradient denotes the probability of bases be paired from high (red) to low (blue). The minimal free energy for folding is given for each structure.

mRNA Tph2-FLAG was found to have different thermodynamic parameters in comparison to mRNA Tph2. Free energy of the thermodynamic ensemble was −488.99 kcal/mol for mRNA Tph2-FLAG vs. −473.16 kcal/mol for mRNA Tph2 and, the ensemble diversity was 412.11 vs. 299.99 for these two ensembles, respectively. Tph2 RNA secondary structure as analyzed in the MFE plot showed a hidden 5′ end start codon in a stem having a strong base-pairing (Fig. 2A). Instead, mRNA Tph2-FLAG has a change in its structure making apparent a free 5′end start codon with low base-pairing probability (Fig. 2B).

### 5-HT quantification in mouse brain

**Fig. 3:**
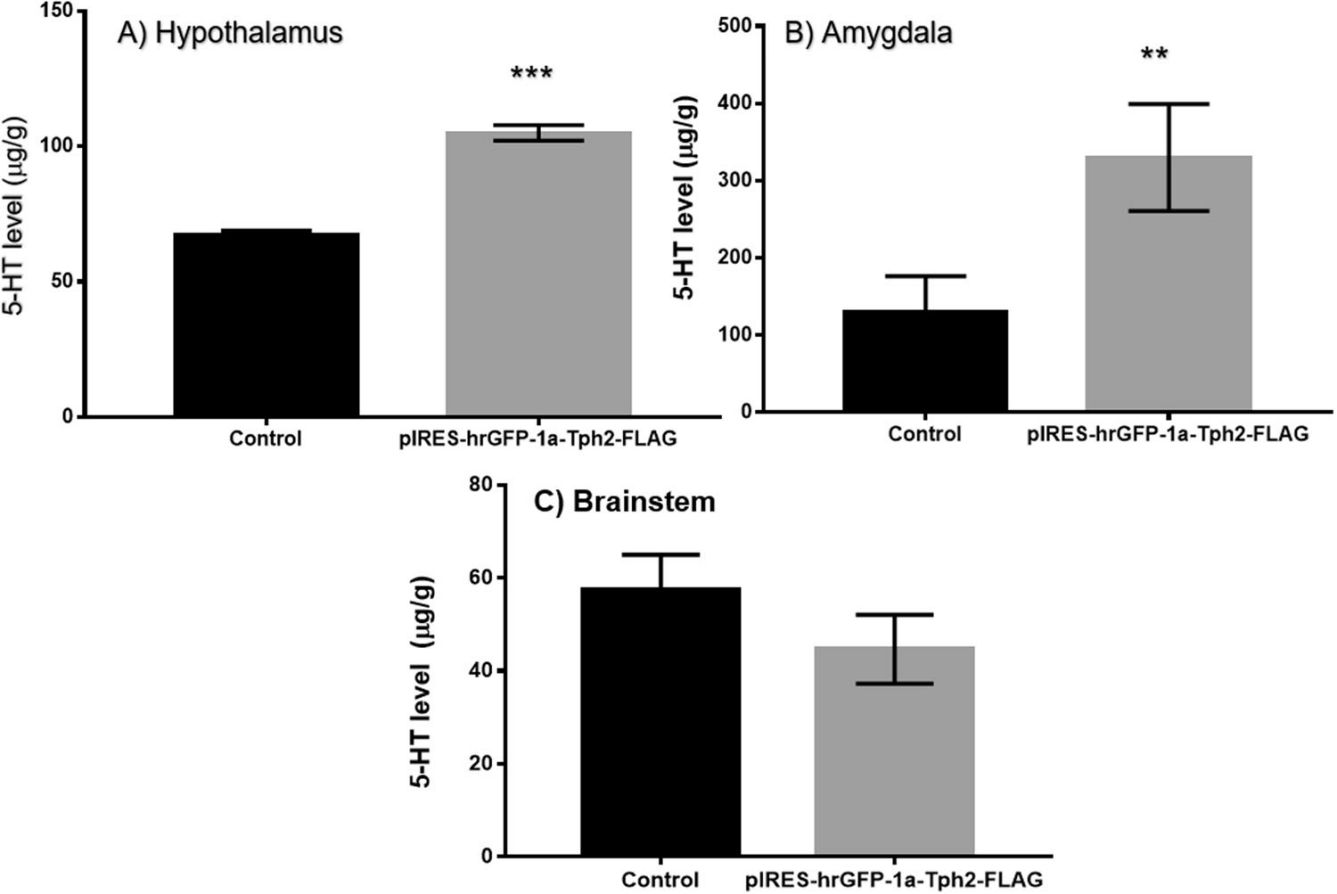
5-HT level in the hypothalamus, amygdala and brainstem of mice following the ocular treatment with pIRES-hrGFP-1a-Tph2-FLAG. Mice treated with the plasmid pIRES-hrGFP-1a-Tph2-FLAG (25 μg) via the ocular route showed significantly higher 5-HT levels than non-transfected control animals both in the amygdala and hypothalamus (**A**, **B**). No changes were observed in the brainstem (**C**). Data are expressed as the mean ± SEM. **P < 0.01, ***P < 0.0001 according to the Student’s *t*-test.

5-HT levels were measured in three brain structures: hypothalamus, amygdala, and brainstem. Ocular administration of the plasmid pIRES-hrGFP-1a-Tph2-FLAG induced a significant increase of 5-HT levels in the amygdala (*P* < 0.01) and hypothalamus (*P* < 0.0001), but not in the brainstem (*P* > 0.05) (Fig. 3).

### Tph2 immunohistochemistry

**Fig. 4:**
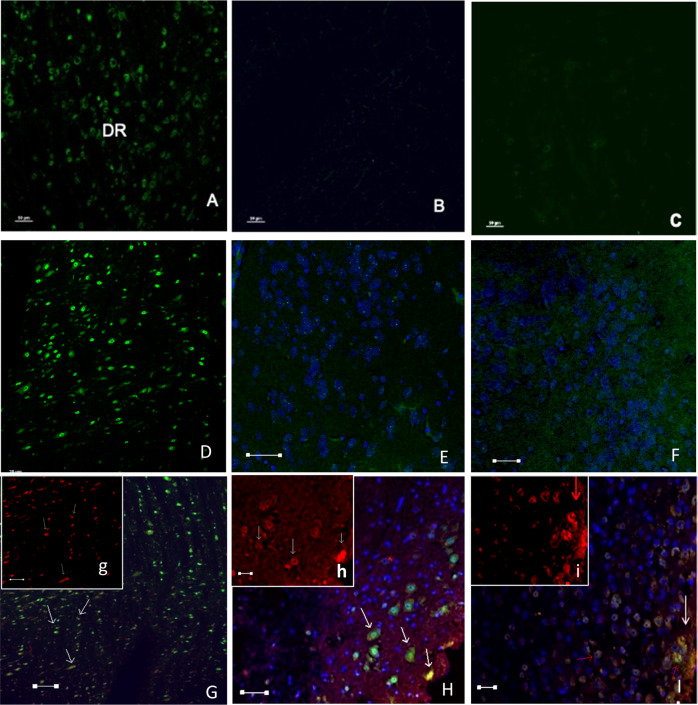
Tph2 and FLAG immunodetection within the brainstem dorsal raphe nucleus, amygdala, and hypothalamus after the ocular administration of pIRES-hrGFP-1a-Tph2-FLAG to mice. Tph2 expression in non-transfected control mice is observed in the cytoplasm of neurons from the dorsal raphe nucleus of the brainstem (**A**) but not in the amygdala (**B**) and hypothalamus (**C**). In transfected animals although Tph2 is also expressed in the cytoplasm of neurons from the dorsal raphe nucleus (**D**) it can also be expressed within the nerve terminals (punctate) in both the amygdala (**E**) and hypothalamus (**F**). Tph2 (green) and FLAG (red) proteins were co-expressed (yellow) (arrows) in both the nuclei and cytoplasm of neurons in the dorsal raphe (**G**; g) and in the nerve terminals of the amygdala (**H**; h) and hypothalamus (**I**; i). Sagittal section. Scale bar: 50 mm in **A**, **D** and **G**; and 20 μm in **B**, **C**, **E**, **F**, **H**, h and f.

Constitutive Tph2 labeling was found in all three brain areas under study. Immunolabeling was however much more intense in the nuclear and cytoplasmic compartments of the pIRES-hrGFP-1a-Tph2-FLAG-treated mice, as compared to the non-treated controls (Fig. 4). Furthermore, a visible number of cells expressing the FLAG sequence was detected in all studied regions of the treated animals. In the brainstem, Tph2 and FLAG proteins were co-expressed in the nuclei and cytoplasm of neurons. In contrast, within the amygdala and hypothalamus, Tph2 and FLAG proteins were mainly co-expressed in the cytoplasm (Fig. 4).

## Discussion

In gene therapy, viral vectors have become the vehicles of choice for many medical applications. Non-viral vectors such as plasmids have also been extensively used to deliver genetic material into host cells due to a number of advantages, including their lack of pathogenicity as well as their low immunogenicity and toxicity [24, 28]. Intranasal and ocular plasmid administration have proved to be excellent non-invasive routes for delivering genetic material into the brain [23, 25].

A non-viral vector was previously described [28] as a good candidate for gene therapy research a few years ago. Very recently, ocular administration of a new construct harboring the murine Tph2 and FLAG-tag sequences (pIRES-hrGFP-1a-Tph2-FLAG) was reported to induce brain expression of recombinant Tph2-FLAG in vivo [25].

In the current contribution, both transfection of HEK-293 cells in vitro and treatment of mice with pIRES-hrGFP-1a-Tph2-FLAG ocularly administered by instillation enhanced the expression of Tph2 as well as the 5-HT levels. The results also suggest that the murine recombinant Tph2-FLAG was also transcribed in the brain mice and was potentially capable of participating in 5-HT biosynthesis.

As can be appreciated, HEK-293 cells without plasmid treatment constitutively produced 5-HT [26, 27], as evidenced by the small 5-HT amounts detected in the lysates from non-transfected cells. However, compared to the control (non-transfected) cells, a significantly higher amount of 5-HT was found in lysates from the HEK-293 cells transfected with pIRES-hrGFP-1a-Tph2-FLAG, suggesting that this construct stimulated 5-HT synthesis. There was the same concentration of 5-HT both in lysates from the control cells and in those transfected with either the vector-only or the vector containing Tph2 alone. Although the reason for the difference in expression between the Tph2 only containing plasmid and that having Tph2-FLAG is difficult to understand it might be explained on the basis of their respective folding properties [29]. Thus, by performing folding analysis and as has been indicating above, Tph2-FLAG RNA secondary structure is both endowed with greater stability than RNA Tph2 owing to a more negative free energy change during folding. In addition, it holds a start codon available for its translation into the Tph2-FLAG enzyme. Under these conditions, it may be conceivable that Tph2-FLAG accumulate easier than Tph2 within the cell allowing the production of higher 5-HT levels. In view of this finding, it will be interesting for future gene therapy work to ascertain whether or not a FLAG molecular tag will help a gene of interest to be more efficiently translated. In support of this hypothesis, it is worth considering that it has been reported that posttranscriptional gene regulation is not only based on the linear sequence of the messenger RNAs but also on their folding into intricate secondary structures. These characteristics, which are highly dynamic and interdependent have been shown to exert direct control over the transcriptome influencing many aspects of cell function [30].

Furthermore, since the recombinant Tph2 was expressed in the brain 72 h following treatment with the plasmid pIRES-hrGFP-1a-Tph2-FLAG [25], the increased 5-HT levels found in the mouse brain and also in HEK-293 cells after transfection may have been generated in both cases by an enhancement of the Tph2 apoenzyme expression rather than by its activation. On this scenery, it may be feasible that the tag FLAG contained in the plasmid used for transfection had only given greater stability to the newly formed enzyme allowing its larger intracellular accumulation.

Particularly interesting is the regional nature of the 5-HT enhancement in the mouse brain herein exhibited following treatment with the pIRES-hrGFP-1a-Tph2-FLAG plasmid. 5-HT levels were significantly higher in the amygdala and hypothalamus of mice treated with pIRES-hrGFP-1a-Tph2-FLAG compared to the same brain regions of control mice. In line with this, both Tph2 and FLAG immunoreactivity were seemingly enhanced within the brainstem suggesting that both parameters bear between them an important relationship. These region-dependent results are in keeping with the anatomical features of the serotonergic system in the brain.

There was, however, no significant difference between groups for the same two parameters in the brainstem. These region-dependent results are in keeping with the anatomical features of the serotonergic system in the brain. Thus, whereas the brainstem contains the raphe nuclei, origen of brain serotonergic innervation, as demonstrated by the pioneering work of Dahlström and Fuxe [31] the nerve terminals of the amygdala and hypothalamus are the places where 5-HT is stored in synaptic vesicles and released on demand.

Although it is unknown how ocularly administered plasmids reach the brain, Lambiase (2007) [32] and Di Fausto (2007) [33] have discussed different routes in order to explain the beneficial effects of the ocular administration of some compounds (e.g., BDNF) capable of reducing the neurodegenerative damage produced in Alzheimer disease (AD) models, which may also account for our results. Accordingly, although our plasmid may reach the brain by a hitherto undiscovered connection between the eye and brain [34, 35], it can also reach it either by diffusion into the cerebral spinal fluid surrounding the optic nerve [31] or by an indirect nasal transport through the nasolacrimal duct and its secondary transport to the brain through the nasal mucosa.

The current contribution provides the first solid evidence of the feasibility of using a non-viral vector administered through a non-invasive route (i.e., topical ocular application) to increase 5-HT levels in brain areas studied in this work, which have been related to depression and anxiety [31]. Although our results are rather encouraging, the main limitations of this work lie in the fact that it is unknown whether the Tph2-FLAG vector is only expressed in serotoninergic neurons and whether the increased 5-HT found after transfection is available to be released on demand. It will remain for the future to study the feasibility of using the approach followed in this work for the treatment of psychiatric patients showing TPH2 polymorphisms associated with depression and suicide attempts in who a decreased 5-HT synthesis may be involved.

## Conclusion

Compared to non-transfected HEK-293 cells and some brain regions from non-treated mice, transfection with the plasmid construct pIRES-hrGFP-1a-Tph2-FLAG results both in vitro and in vivo in an increase of 5-HT levels most probably due to an enhancement in its biosynthesis. Furthermore, although our results are suggestive, further research will be needed on the possibility of employing pIRES-hrGFP-1a-Tph2-FLAG as a treatment for psychiatric diseases (e.g., major depression) involving a Tph2 dysfunction.

## Supplementary information

Figure legends
