# Supplementary material for: Increase of 5-HT levels is induced both in mouse brain and HEK-293 cells following their exposure to a non-viral tryptophan hydroxylase construct
Source: Transl Psychiatry. 2021 Oct 8;11:515. doi: 10.1038/s41398-021-01634-x (PMC8501106; doi:10.1038/s41398-021-01634-x)
Supplement: Supplementary file 1 — Figure legends [file 41398_2021_1634_MOESM1_ESM.docx]

**Figure legends**

**Figure 1. 5-HT level in HEK-293 cells.**

Serotonin levels were measured in HEK-293 cells by ELISA at 48 h post-transfection. Evaluation was made in supernatants from lysates of both non-transfected (control) cells and from cells transfected with any one of the following three different treatments: pIRES (pIRES-hrGFP-1a), a commercial plasmid only having the vector; (pIRES-hrGFP-1a-Tph2), the murine *Tph2* gene construct without FLAG and (pIRES-hrGFP-1a-Tph2-FLAG), the murine *Tph2* gene construct with the FLAG tag. Cell lysates following transfection were centrifuged and their supernatants used to measure 5-HT. The statistical analysis shows a significantly greater 5-HT content in the lysates from cells treated with the construct pIRES-hrGFP-1a-Tph2-FLAG as compared to the lysates from the cells of all the other groups. Results are expressed as Means ± SEM, One-way ANOVA: F: 40.48, ***P < 0.0001.

**Figure 2.** **Tph2 RNA secundary structure.**

A) Representation of the murine Tph2 mRNA folding showing its wild type 3' end. A red arrow points out to the start codon in the 5' end. The start codon is structured in a stem that could prevent its proper translation into protein.

B) Tph2-FLAG mRNA secundary structure shows the modified 3' end by the fusion of two FLAG sequences, which breaks the stem structure that contained the 5' AUG codon.

A red arrow indicates the 5' end of the molecule that allows the start codon to be accessible to the ribosomes.

Color gradient denotes the probability of bases be paired from high (red) to low (blue). The minimal free energy for folding is given for each structure.

**Figure 3. 5-HT level in the hypothalamus, amygdala and brainstem of mice following the ocular treatment with pIRES-hrGFP-1a-Tph2-FLAG.**

Mice treated with the plasmid pIRES-hrGFP-1a-Tph2-FLAG (25 µg) via the ocular route showed significantly higher 5-HT levels than non-transfected control animals both in the amygdala and hypothalamus. No changes were, however, observed in the brainstem (ns). Data are expressed as the mean ± SEM. **P< 0.01 ***P < 0.0001 according to the Student’s *t*-test.

**Figure 4. Tph2 and FLAG immunodetection within the brainstem dorsal raphe nucleus, amygdala and hypothalamus after the ocular administration of pIRES-hrGFP-1a-Tph2-FLAG to mice.**

Tph2 expression in non-transfected control mice is observed in the cytoplasm of neurons from the dorsal raphe nucleus of the brain stem (A) but not in the amygdala (B) and hypothalamus (C). In transfected animals although Tph2 is also expressed in the cytoplasm of neurons from the dorsal raphe nucleus (D) it can also be expressed within the nerve terminals (punctate) in both the amygdala (E) and hypothalamus (F). Tph2 (green) and FLAG (red) proteins were co-expressed (yellow) (arrows) in both the nuclei and cytoplasm of neurons in the dorsal raphe (G; g) and in the nerve terminals of the amygdala (H; h) and hypothalamus (I; i). Sagittal section. Scale bar: 50 mm in A, D and G; and 20 μm in B, C, E, F, H, h and f.
